# Genetic Variability and Population Divergence in the Rare *Fritillaria tubiformis* subsp. *moggridgei* Rix (Liliaceae) as Revealed by RAPD Analysis

**DOI:** 10.1371/journal.pone.0101967

**Published:** 2014-07-15

**Authors:** Marco Mucciarelli, Diana Ferrazzini, Piero Belletti

**Affiliations:** 1 Department of Life Sciences and Systems Biology, University of Torino, Torino, Italy; 2 Department of Agricultural, Forest and Food Science, University of Torino, Grugliasco (Torino), Italy; University of Milano Bicocca, Italy

## Abstract

*Fritillaria tubiformis* subsp. *moggridgei* Rix. is a rare alpine geophyte with shiny yellow flowers. This plant is sporadically distributed across the southwestern Alps where it is biogeographically close to *F. tubiformis* var. *burnatii* Planch. The latter has dark purple flowers and ranges in the majority of the Western and Central Alps. In order to develop appropriate strategies of conservation, a RAPD based analysis was conducted to study the genetic status of these taxa and the distribution of genetic variability of the subspecies by sampling seven populations distributed across the subspecies' range. Four populations of var. *burnatii* were chosen within this range and included in the genetic analysis. Some 264 individuals were analysed and 201 polymorphic loci were scored. Genetic diversity scored in the subspecies was in line with expectations for endemic species (H_e_  = 0.194). *F. tubiformis* var. *burnatii* showed lower intraspecific diversity (H_e_  = 0.173), notwithstanding a wider range than the subspecies. Most of the total phenotypic variation (about 83%) was allocated within populations, and significant lower proportions between taxa (6.45%) and between populations of the same taxon (10.64%). Moreover, PCoA analysis and Bayesian clustering separated populations into two genetically differentiated groups corresponding with the subspecific taxa. However, three populations ascribed to the subsp. *moggridgei* repeatedly showed genetic admixture with var. *burnatii* populations. Our findings suggest that: i) although the different flower colour, the two taxa are genetically very similar and share a consistent part of their gene pool, ii) the majority of genetic variability is allocated within populations rather than among them, iii) a representative amount of genetic diversity can be preserved by sampling from a restricted number of populations. The efficacy of RAPD markers in analysing genetic variation, and the contribution of the results to the preservation of biodiversity of the species, are discussed.

## Introduction

Genetic erosion is one of the most important threats to the survival of many wild species. Nowadays these species are subjected to many stress factors, most of which are due to human activities: namely environment alteration, habitat fragmentation, urbanisation, pollution, irrational forest management and the introduction of allochthonous species. Current climate changes will probably determine a decline in growth and survival, and an alteration of genetic structure and diversity for many wild species. This pressure may be strengthened in marginal and peripheral populations of the species distribution, mainly in European southern populations of plants [1]. In order to survive these threats, and to persist over time, a high adaptive potential is fundamental: this is mainly determined by the within-species genetic diversity [2]. Genetic differentiation derives from several evolutionary forces, among which selection pressure as a response to different ecological conditions and genetic drift are the most important. Gene flow, in contrast to this pattern, leads to homogenisation of allelic frequencies [3].

The characterisation of patterns of genetic diversity within species, and among populations, is a fundamental necessity to establish any program aimed at preservation of biodiversity. Genetic markers are basic for supplying information on the genetic structure of populations, to analyse the pattern of within-species variability distribution [4] and to support the management of seed supply [5]. In particular, knowledge of genetic variation should be the basis for actions of ecological restoration [6]. This is even more important where the preservation of local, marginal and probably well adapted and/or differentiated gene pools must be achieved, through the management of small, marginal and inbreed populations [7], [8]. As a consequence, the goal is to focus not only on scattered and rare species, but also on those characterised by wide distribution that could appear to be less sensitive to genetic erosion [9].

*Fritillaria tubiformis* subsp. *moggridgei* (Boiss. & Reut. ex Planchon) Rix (Liliaceae) is a rare taxon sporadically distributed along the Ligurian and Maritime Alps. The species is limited to a few main sites within the Parc National du Mercantour in France (Muséum National d'Histoire Naturelle. National inventory of natural heritage. Website: http://inpn.mnhn.fr [accessed 17 04 2014]), and to seven populations of no more than 1000–10000 individuals found across Piedmont and Liguria, in Italy.

Conversely, *Fritillaria tubiformis* Gren. var. *burnatii* Planch. has a wider range, including the majority of the Western and Central Alps [10]. In the past, the two taxa have been confused and often fully synonymised by Italian botanists with *F. tubiformis* Gren. & Godr. [11], from which they differ by leaf morphology and the colour of tepals, which in *moggridgei* are yellow instead of dark purple. Results of a phylogenetic analysis carried on combined DNA sequences of the partial *matK* gene and the *rpl16* intron supported this view, and showed that subsp. *moggridgei* is genetically more closely related to var. *burnatii* than to any other species in the genus. However, in the same study, using plastid length-variable microsatellites a number of plastid haplotypes were found to be differently fixed in the two subspecies [12].

Owing to this taxonomic uncertainty, our genetic analysis has been focused on the seven Italian populations of subsp. *moggridgei*. In addition, we have used a data set based on DNA from four populations of *F. tubiformis* var. *burnatii*, sampled at the periphery of the subspecies distribution. A biosystematic study is also underway aimed at clarifying the exact phylogenetic relationships between the two taxa and their distribution range.

Random Amplified Polymorphic DNA (RAPD) technology is based on the amplification, using the Polymerase Chain Reaction (PCR), of short segments of DNA randomly chosen by the use of arbitrary, usually short (8–12 base pairs) primers. Despite having some drawbacks, the most important of which are the dominant expression and the low level of reproducibility [13], [14], the RAPD technique analyses a larger number of loci, and thus provides a more general evaluation of the genome with a high potential for detecting polymorphism. Furthermore, the technique has a low cost, and can give a large amount of data in a short time; it requires small amounts of DNA and there is no requirement of prior knowledge of the genome being studied.

The aims of the present research were to estimate the amount and distribution of genetic diversity within and among *Fritillaria tubiformis* subsp. *moggridgei* populations from north-western Italy using RAPDs, and to evaluate possible effects of habitat fragmentation on genetic variation. The genetic relationship between the subspecies and *Fritillaria tubiformis* var. *burnatii* has also been investigated. Spatial structure and the expected association between genetic and geographical distances among populations were assessed, in order to provide recommendations for in situ preservation of the species.

## Materials and Methods

### Plant material

**Figure 1:**
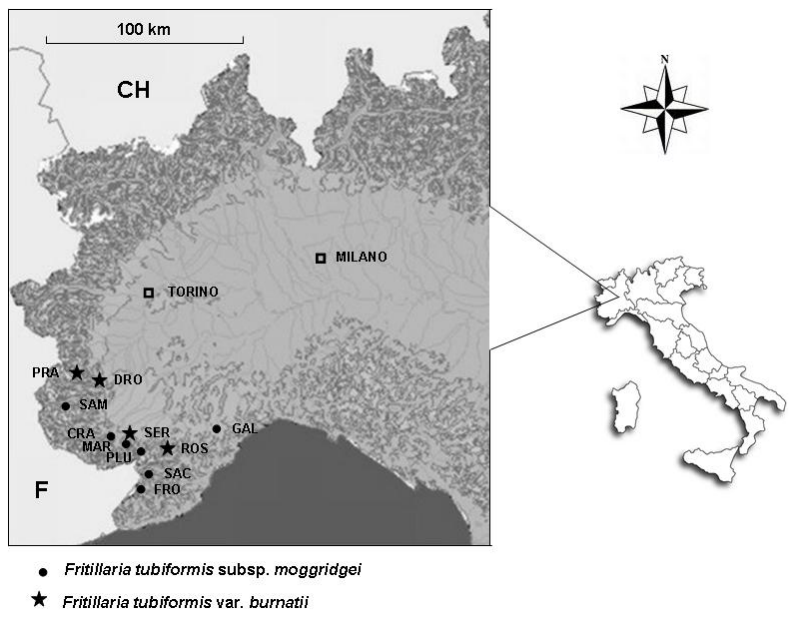
Geographical distribution of the Italian populations of *Fritillaria tubiformis* subsp. *moggridgei* (circles) and *Fritillaria tubiformis* var. *burnatii* (stars) analysed in this study.

Seven populations of *F. tubiformis* subsp. *moggridgei* were considered in the study, distributed across the Maritime and Ligurian Alps, which represent the natural range of the subspecies in Italy. In general, populations were of small size and individuals therein limited to several hundreds. In addition, four populations of *F. t.* var. *burnatii* were also considered and were sampled within an area that partly overlaps the distribution range of the former subspecies. Figure 1 and Table 1 summarise names and locations of the populations analysed. Ente di gestione del Parco naturale del Marguareis (Chiusa di Pesio, province of CN) issued the permit for leaf sampling within PLU, MAR and SER populations (see Table 1 for their location). For the remaining locations (Table 1) specific permission was not required since these locations are outside protected areas and leaf collection does not represent a threat to the sampled individuals.

**Table 1:** Details of the site characteristics of *Fritillaria tubiformis* populations from Italy which were sampled for the study.

Taxon*	Code	Locality	Location	Elevation (m a.s.l.)
M	SAM	Sambuco (Stura Valley)	44° 20′ N–7° 05′ E	1430
M	CRA	Cravina (Pesio Valley)	44° 14′ N–7° 39′ E	1420–1540
M	MAR	Marguareis (Pesio Valley)	44° 11′ N–7° 40′ E	1430
M	PLU	Pian del Lupo (Pesio Valley)	44° 11′ N–7° 41′ E	1990
M	SAC	Saccarello (Tanaro Valley)	44° 04′ N–7° 43′ E	1900
M	FRO	Frontè (Tanaro Valley)	44° 03′ N–7° 45′ E	1890–1980
M	GAL	Galero (Tanaro Valley)	44° 09′ N–8° 00′ E	1690
B	PRA	Prazzo (Maira Valley)	44° 31′ N–7° 02′ E	1960–2020
B	DRO	Droneretto (Maira Valley)	44° 31′ N–7° 12′ E	1700–1800
B	SER	Serpentera (Pesio Valley)	44° 13′ N–7° 41′ E	1890–2000
B	ROS	Pian Rosso (Tanaro Valley)	44° 09′ N–7° 46′ E	1800–1910

*M =  Fritillaria tubiformis subsp. moggridgei, B =  Fritillaria tubiformis var. burnatii.

Leaves were sampled from about 30 non-adjacent blooming individuals in each population, in order to avoid selection of clonal individuals. In total, 264 individuals were analysed. After collection, the vegetative material was frozen at −20°C until DNA extraction.

### Molecular analysis

Total DNA was extracted from frozen tissue using E.Z.N.A. SP Plant DNA Kit (Omega Bio-Tek, Norcross, GA), and purified on columns following the manufacturer's protocol. Amplifications were carried out in a Perkin Elmer DNA thermal cycler (PE 9600). The optimal reaction for RAPD analysis was set using the following conditions: 25 µl volume containing 1× reaction buffer, 1 unit of Taq DNA polymerase (Promega), 2.5 mM magnesium chloride, 0.2 µM decamer primers, dNTPs mix 0.2 mM and 20 ng DNA. The amplification conditions were as follows: the first step at 95°C for 5 min., followed by 40 cycles of 1 min. at 94°C, 1 min. at 36°C and 2 min. at 72°C. The reaction was completed with a final run at 72°C for 8 min. We screened a total of 16 primers from Life Technologies. The amplification products were separated in 1.5% agarose gel with ethidium bromide in 0.5× TBE buffer. The banding patterns were visualised under UV light and acquired using a fluorimeter linked with a gel documentation system (GelDoc 2000). The bands were scored visually, and those of similar molecular size, scored for the same primer, were assumed to be homologous. Since RAPD markers are dominant it was assumed that each band was an independent locus with two alleles: presence or absence of the band. The reproducibility of the amplification products was tested twice for each sample and each primer.

### Data processing

Amplified fragments were scored as presence or absence of bands, and a binary matrix of RAPD phenotypes was assembled. Only polymorphic loci were used. The data analysis were further restricted to bands whose observed frequencies in each population were less than 1 – (3/N), where N is the number of plants analysed [14] to avoid significant bias in estimates of population genetic parameters. Non-random associations between pairs of loci were investigated using the Spearman rank correlation, with the SPSS 12.0 computer package. Intrapopulational genetic diversity was assessed as the proportion of polymorphic loci (P, using the 95% criterion), mean number (N) and effective number (N_e_) of alleles per locus, Nei's gene diversity [15] (H_e_, that was adopted assuming the populations to be in Hardy-Weinberg equilibrium, although we were not able to investigate this since dominant markers were used), and Shannon's information index. The latter can be considered a measure of phenotypic diversity (I =  −Σp_i_log_2_p_i_, where p_i_ is the frequency of presence or absence of a given RAPD fragment; [16]), assuming that populations are not in Hardy-Weinberg equilibrium. This index is frequently used in RAPD analysis because it is insensitive to bias that may be introduced into data owing to undetectable heterozygosity [17]. Calculations were performed using the GENALEX 6.5 software package [18].

The distribution of genetic diversity within and among populations was assessed using Nei's genetic differentiation degree (G_ST_) [19]. Analysis of Molecular Variance (AMOVA), based on squared Euclidean distances between all pairs of RAPD phenotypes [20], was employed using the Arlequin software [21]. The AMOVA procedure was performed in order to further partition the total genetic variation among taxa, among population within subspecies and within populations, and to compute a pairwise population F_ST_ value matrix according to Weir and Cockerham [22]. The statistical significance of the covariance components was estimated by nonparametric randomisation tests using 10000 permutations. The null distribution of pairwise F_ST_ values under the hypothesis of no differences between the populations was also tested by using a permutational approach (10000 replicates).

Cluster analysis was performed on pairwise F_ST_ distances using the Unweighted Pair Group Method with Arithmetic Averages (UPGMA; [23]) with the SAHN program in NTSYS-pc 2.10j [24]. A cophenetic value matrix (COPH in NTSYS) was produced from the dendrogram and compared with the genetic distance matrix by using the MXCOMP program in NTSYS to estimate the goodness of fit of the cluster analysis. Principal coordinate analysis (PCoA) was also performed, based on pairwise F_ST_ distances matrix (DCENTER and EIGEN procedures in NTSYS), to better understand genetic relationships among populations.

The genetic structure of the populations was also explored using Bayesian clustering, performed using the software Structure [25]. The program uses a Markov chain Monte Carlo (MCMC) algorithm to cluster individuals into populations on the basis of multilocus genotypic data. Individual multilocus genotypes are first assigned probabilistically to genetic clusters (K) without considering sampling origins. Admixed or hybrid individuals can be identified as they will have a fraction of their alleles derived from each genetic cluster. Posterior probabilities of K were calculated from the means of 20 runs for each value of K (from 1 to 6), and the optimum K determined using the method of Evanno et al. [26].

## Results

### Variation of RAPD loci

Among the primers tested, OPA01, OPA02, OPA03, OPA4, OPA6, OPAF04, OPAF12 and OPAS18 gave non-reproducible amplification products and were therefore excluded from the analysis. The remaining eight selected primers showed reliable banding patterns and generated a total of 204 consistent and differential amplification products, ranging in size from 170 to 990 bp. Each primer amplified between 15 (OPA10) and 30 (OPA19) fragments, with a mean number of scored bands per primer of 25.5 (Table 2). Almost all these bands were polymorphic, being monomorphic only three, OPA10-4, OPA12-1 and OPA19-1. Many of the bands were fixed at the population level, and several even at the subspecific level (16 within subsp. *moggridgei* and 4 within var. *burnatii*). Five bands were unique to a single population: OPA05-19, OPA05-30, OPA05-21 and OPA09-27 in GAL, OPA05-22 in ROS. All bands fulfilled the Lynch and Milligan criterion [14], and were therefore included in further analyses. No band pair showed any correlation with any other, as indicated by Spearman's rho correlation coefficients (data not showed). Therefore, each of the 201 polymorphic bands was considered as an independent locus.

**Table 2:** Characteristics of fragments generated by the 8 primers selected for the genetic analysis.

Primer	Nucleotide sequence (5′ → 3′)	Molecular weight range (bp)	Total number of bands	Number of polymorphic bands
OPA05	AGGGGTCTTG	190–930	26	26
OPA07	GAAACGGGTG	170–940	28	28
OPA09	GGGTAACGCC	180–700	27	27
OPA10	GTGATCGCAG	340–920	15	14
OPA12	TCGGCGATAG	280–990	26	25
OPA15	TTCCGAACCC	210–920	29	29
OPA18	AGGTGACCGT	200–850	23	23
OPA19	CAAACGTCGG	240–870	30	29
**Total**	**-**	**-**	**204**	**201**

### Genetic variation within populations

Genetic variation within populations was estimated for the 11 populations using various methods (Table 3). The mean number of alleles per locus ranged from 1.48 (PRA) to 1.72 (GAL), with a mean value of 1.59 (standard error, SE  = 0.017). The mean effective number of alleles per locus was 1.29 (SE  = 0.006), and the range was from 1.25 (ROS) to 1.32 (MAR). The percentage of polymorphic loci ranged from 72 (PRA) to 85 (GAL), with a mean value of 77.5 (SE  = 1.19), and the expected heterozygosity was on average 0.187 (SE  = 0.003), ranging from 0.165 (ROS) to 0.206 (CRA). Lastly, the Shannon diversity index, based on phenotype frequency, ranged from 0.268 (ROS) to 0.327 (GAL), with a general mean of 0.299 (SE  = 0.005). In general, populations belonging to *Fritillaria tubiformis* subsp. *moggridgei* showed an amount of internal genetic variability slightly higher than those of *Fritillaria tubiformis* var. *burnatii*.

**Table 3:** Genetic variation within populations of *Fritillaria tubiformis* from Italy, based on 201 RAPD markers.

Population	Taxon*	S	N	N_e_	P	H_e_	I
SAM	M	24	1.63	1.26	79	0.173	0.284
CRA	M	24	1.64	1.33	80	0.206	0.327
MAR	M	24	1.55	1.32	75	0.203	0.318
PLU	M	24	1.62	1.31	79	0.196	0.312
SAC	M	24	1.58	1.28	77	0.183	0.295
FRO	M	24	1.66	1.31	81	0.198	0.318
GAL	M	24	1.72	1.30	85	0.199	0.322
PRA	B	24	1.48	1.28	72	0.179	0.284
DRO	B	24	1.55	1.28	75	0.178	0.285
SER	B	24	1.56	1.26	76	0.171	0.278
ROS	B	24	1.49	1.25	73	0.165	0.268
**General mean**		**264**	**1.59 (0.017)**	**1.29 (0.006)**	**77.5 (1.19)**	**0.187 (0.003)**	**0.299 (0.005)**
*F. t. moggridgei*	M	168	1.63 (0.016)	1.30 (0.006)	79.4 (1.22)	0.194 (0.004)	0.311 (0.006)
*F. t. burnatii*	B	96	1.52 (0.019)	1.27 (0.006)	74.0 (1.20)	0.173 (0.003)	0.279 (0.006)

*M =  Fritillaria tubiformis subsp. moggridgei, B =  Fritillaria tubiformis var. burnatii.

S =  sample size, N =  mean number of alleles per locus, N_e_  =  effective number of alleles per locus, P =  percentage of polymorphic loci (95% criterion), H_e_  =  Nei's (1978) unbiased expected gene diversity, I =  Shannon's index over loci. Standard errors are given in parenthesis.

### Genetic variation among populations and groups

Partitioning of the total gene diversity (H_T_  = 0.293), following Nei's statistics [19], revealed that about 12.9% of the total variation was allocated among taxa and 4.5% among populations belonging to the same taxon. The remaining genetic variation was due to differences between individuals within populations. Within taxa, *F. tubiformis* subsp. *moggridgei* showed a genetic differentiation slightly higher than *F. tubiformis* var. *burnatii* (values of G_ST_ respectively of 0.135 and 0.117). The hierarchical analysis of molecular variance (AMOVA) confirmed these findings, demonstrating that about 83% of the variation was within populations and the remaining variance was attributable partly to differences between groups (6.45%) and partly among populations (10.64%), providing corroborating evidence for the genetic structure predicted by the Nei's indices analysis (Table 4).

**Table 4:** Analysis of molecular variance (AMOVA) within and among the populations studied.

Source of variation	df	Sum of squares	Variance components	Percentage of variation	P
Among groups	1	357.245	2.046	6.45	<0.001
Among population within groups	9	965.217	3.373	10.64	<0.001
Within populations	253	6.651.667	26.291	82.91	<0.001

**Figure 2:**
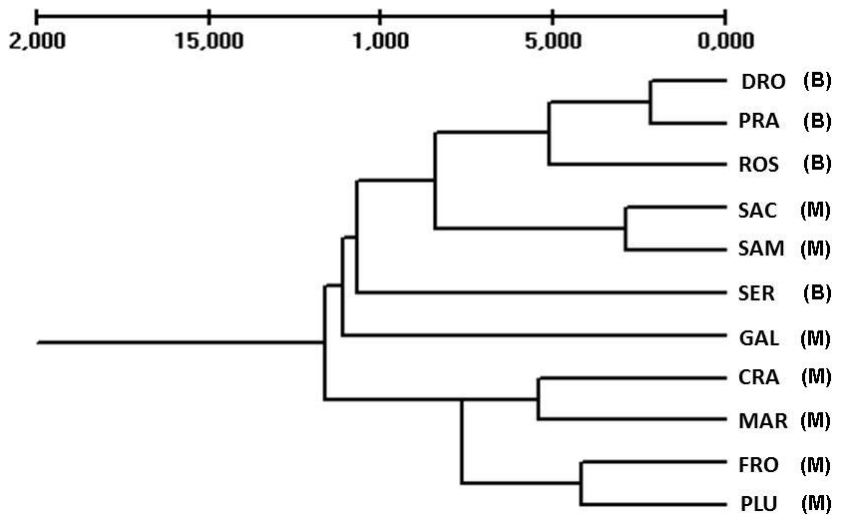
Dendrogram based on UPGMA of RAPDs profiles, showing relationships among the 11 populations of *Fritillaria tubiformis* considered in the study. M =  *Fritillaria tubiformis* subsp. *moggridgei*, B =  *Fritillaria tubiformis* var. *burnatii*.

Cluster analysis, based on pairwise F_ST_ distances between all pairwise combinations of individuals, was used to generate an UPGMA dendrogram in order to examine relationships between populations (Figure 2). Populations clustered thoroughly in two distinct main groups, showing low geographical correspondence. The first cluster contained all the four populations of var. *burnatii* but with intermixed populations of subsp. *moggridgei*. The DRO and PRA populations (both sampled in Maira Valley) clustered together as a sister group to ROS (Tanaro Valley) but also to SAC and SAM belonging to subsp. *moggridgei*. The second cluster grouped together the remaining populations belonging to subsp. *moggridgei* and precisely CRA and MAR (from Pesio Valley) as a sister group to the populations FRO and PLU. No precise assignation was possible for the remaining two populations, SER and GAL, belonging respectively to var. *burnatii* and subsp. *moggridgei*.

**Figure 3:**
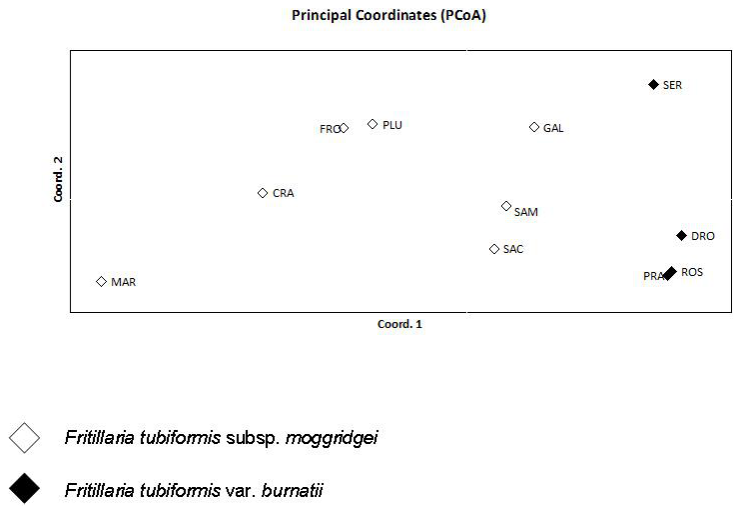
PCoA plot based on RAPDs variation, with the first two principal components showing relationships among the 11 populations of *Fritillaria tubiformis* studied.

The PCoA analysis was performed to visualise the grouping of populations more clearly, providing a graphical representation of the relationships between them (Figure 3). Calculated pairwise Euclidean distances were used as an input for PCoA. The first two principal coordinate axes accounted for 49.76% and 21.35% of the total variation (cumulative value 71.11%). The first principal coordinate clearly distinguished subspecific taxa, but when referred to the second coordinate, populations were broadly scattered and clustered independently of each other (Figure 3).

**Figure 4:**
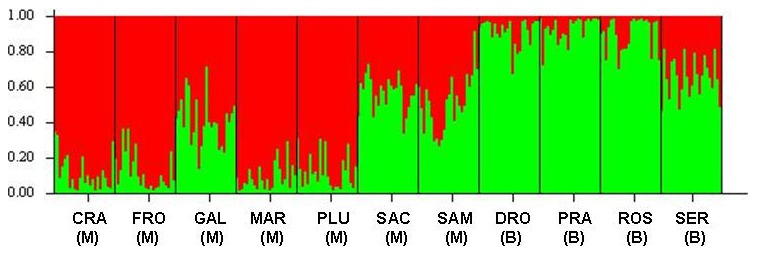
Probability of assignment of 264 plants of *Fritillaria tubiformis* from Italy to the two genetic clusters identified by hierarchical STRUCTURE analysis, and corresponding with subspecific taxa. Each vertical bar corresponds with a distinct genotype and different colours indicate the part of its genome assigned to each cluster. M =  *Fritillaria tubiformis* subsp. *moggridgei*, B =  *Fritillaria tubiformis* var. *burnatii*.

Following the method of Evanno et al. [26], the Bayesian clustering results obtained with STRUCTURE indicate that K = 2 clusters represents the most informative representation of the overall genetic structure that we analysed (Figure 4). We found that most individuals from subsp. *moggridgei* (CRA, FRO, GAL, MAR, and PLU) clearly belong to cluster 1, whereas var. *burnatii* populations (DRO, PRA, ROS and SER) are primarily composed by individuals from cluster 2. Exceptions are represented by two admixed populations belonging to subsp. *moggridgei* (SAC and SAM), that already in the UPGMA analysis were shown to be more similar to var. *burnatii* than to other populations of the same taxon.

## Discussion

This study investigated genetic variation of *Fritillaria tubiformis* subsp. *moggridgei* endemic of the South-Western Alps. Genetic structure in this subspecies was compared with four populations of *Fritillaria tubiformis* var. *burnatii* sampled within the range of the subspecies.

RAPD markers proved to be an efficient tool to analyse genetic variation both within and among populations. However, it must be underlined that RAPD variation cannot be linked with variation in adaptive genes [27], so that further research is needed to better understand the relationship between the observed variation and the adaptive potential of these taxa. The use of RAPD markers to estimate genetic parameters in outcrossing populations has been questioned: since RAPDs are dominant markers, null alleles present only in heterozygous genotypes cannot be detected, thus underestimating gene diversity [14].

In species with complex life histories, the choice of appropriate techniques of genetic analysis is not obvious. In a previous analysis run on chloroplast DNA with different type of sequence repeats, we showed that only insertion/deletion events were effective in discriminating genetically between yellow and dark purple flowered-morphotypes of *F. tubiformis* [12]. In the present study, RAPDs amplified mostly non-coding nuclear DNA sequences, which are subject to weaker selection pressures, thereby allowing the scoring of a higher amount of genetic variation.

*F. tubiformi*s subsp. *moggridgei* displayed a level of genetic diversity in line with expectations. Expected heterozygosity (H_e_  = 0.194) (Table 3) was similar to the average value of H_pop_ reported by Nybom [28] for endemic species (0.20), and for species with seeds dispersed by gravity (H_pop_  = 0.19).

The average percentage of polymorphic loci (75–85%) (Table 3) and total gene diversity (0.293) were in the range previously reported with RAPDs for other endemic or endangered species [17], [29], [30], [31]; in contrast, percent polymorphism was higher in subsp. *moggridgei* than in the Iranian endemic *F. imperialis* L. a much more widespread congeneric [32].

Despite the relatively restricted geographical range covered by this investigation, *F. tubiformi*s populations exhibited genetic divergence. Using the AMOVA procedure, we showed that although most of the phenotypic variation was allocated within populations (82.91%), a significant proportion was attributable to genetic differences between intraspecific taxa (6.45%), as well as between populations (10.64%) (Table 4).

The overall F_ST_ value (0.171) was significantly different from zero (P<0.001) (Table 4), providing evidence for a strong genetic structuring among populations. This pattern of differentiation is expected for species pollinated by insects, and with scattered distribution. In this case genetic differentiation is generally higher than that occurring in wind-pollinated species with a more continuous distribution [33], [34]. Significant levels of variation among populations were also observed by partitioning of variability based on Nei's gene diversity (G_ST_  = 17.4%), confirming the existence of a genetic structuring among populations.

The results also indicate that most of the total genetic variation was present within populations. Patterns of high genetic diversity at both the species' and local population levels have already been documented in other mountain plants [35] and critically endangered species [36], [37], especially when relatively large populations have been considered [38]. Our findings, however, were in contrast with two studies on *F. imperialis* and *F. camschatcensis* using RAPD markers, where variation within populations accounted for only 54% and 35.7% of the total variation, respectively [32], [39]. As stressed in the study with chloroplast DNA, when molecular variation is based on allele frequencies, a slight over-estimation of the within population component of variation is possible [12]. This is likely to have occurred in the present study. Moreover, other ecological and demographical characteristics are likely to have promoted the maintenance of high levels of genetic variation in subsp. *moggridgei* populations. This is supported by previous RAPD-based analyses which showed that long-lived, outcrossing or late successional species retain most of their genetic variability within populations [35], [40]; and by contrast, annual, selfing and/or early successional plants allocate most of the genetic variability among populations [28]. The results stress that *F. tubiformis* subsp. *moggridgei* has not experienced population bottlenecks, and the general low levels of inbreeding likewise suggest a predominantly outcrossing mating system for this taxon [40], in accordance with field surveys and breeding features of related species [41].

Population size and geographical separation may be strong determinants of the spatial distribution of genetic variation in populations of neighboring species with fragmented distributions [42]–[43]. These conditions, in combination with life-history features such as growth form, mating system and seed dispersal mechanisms [44], may have conditioned the genetic history of *F. tubiformis* subsp. *moggridgei* populations. SAM, for example, has little chance of gene flow. This is in fact a very small population at a relatively low altitude, which has suffered the impact of agricultural activities and human settlements. GAL and SAC populations, on the contrary, are isolated populations, which nevertheless might receive frequent visits of pollinators thanks to their position on the very summit of two mountains.

Analyses of population genetic structure (UPGMA, PCoA and Structure) showed that the studied populations belonged to two distinct genetic pools corresponding to the subspecific taxa. The varied geological, topographical and climatic conditions of the Alpine chain can boost isolation and divergence of populations even over short geographical distances, thus explaining the fragmented distribution and genetic complexity of some endemic species of the Maritime Alps [45]. This genetic separation, however, was never rigorous, and three populations of subsp. *moggridgei* showed repeated admixture with var. *burnatii*, leaving open the problem of their exact taxonomic attribution. The profile of the UPGMA tree supported a certain degree of genetic admixture across SAC and SAM populations, which positioned in the tree within populations of var. *burnatii* (Figure 2). When analyzed by Structure, genetic admixture was present to some extent also in GAL population (Figure 4). These findings are in accordance with a previous phylogenetic analysis, where we showed that subsp. *moggridgei* was more phylogenetically related to var. *burnatii* (*matK* and *rpl1*6 genes) than to any other *Fritillaria* species considered in the study [12].

If the ambiguous positioning of GAL population in these analysis can be explained by its geographical position and low demographic size, no explanations seems possible for the other two populations. Moreover, clustering of apparently distant populations as SAC (Val Tanaro, Ligury) and SAM (Val Stura, Piedmont) demonstrated that population positioning in the UPGMA tree was almost geographically unrelated. In support of this finding, it has been observed that when analysed by RAPD markers, population diversity is little affected by geographical range [28]. The UPGMA results were supported by PCoA that showed that populations were distributed along the first coordinate according to the intraspecific taxa (almost 50% variation), but showed admixture according to the second coordinate (SAC and SAM in subspecies *moggridgei* and SER in the variety *burnatii*) (Figure 3).

The position of the three populations of subsp. *moggridgei* is ambiguous and it remains unclear whether they are a hybrid of recent origin between subsp. *moggridgei* and var. *burnatii* [46] or whether they represent a separate evolutionary lineage. The low levels of genetic divergence among populations encountered in this study, seem to support the first hypothesis. However, broader taxon sampling in the territory of France might shed more light on this interesting problem.

*F. tubiformis* sensu lato is increasingly rare in the wild, due to habitat loss, and some populations are already under threat because of human activities. In conclusion, this study has shown that *F. tubiformis* subsp. *moggridgei* has a substantial degree of genetic diversity, which may counteract genetic erosion and maintain high adaptive potential in the subspecies. Indeed, this potential is mainly determined by the species genetic diversity [2]. The results show that despite being a narrow endemic with a relatively small distribution and fragmented, isolated populations, *F. tubiformis* subsp. *moggridgei* exhibits high level of genetic diversity and, in the perspective of a long-term conservation, a restricted number of its populations would be variable enough to represent the genetic variability of the subspecies.

A literature and herbarium-based survey of subsp. *moggridgei* and var. *burnatii* has shown that the taxonomic status of the Italian populations is uncertain [11]. A thorough phylogenetic analysis aiming at assessing their genetic relationships with respect to other widespread congeneric taxa of the Alpine chain (for example *F. tubiformis* Gren. & Godr.), will provide information essential for the formulation of appropriate management strategies for the conservation of all these populations.

## Supporting Information

Dataset S1 ***Fritillaria tubiformis*** **subsp.** ***moggridgei*** **RAPD profile and Analysis of Molecular Variance (AMOVA).** The pairwise *F*_ST_ matrix of all population comparisons and step by step pairwise distances along with population labels are also given and can be used to manually calculate AMOVA results.
(XLSX)
